# Study protocol for the COvid-19 Toolbox for All IslaNd (CONTAIN) project: A cross-border analysis in Ireland to disentangle psychological, behavioural, media and governmental responses to COVID-19

**DOI:** 10.12688/hrbopenres.13105.2

**Published:** 2021-02-15

**Authors:** Catherine D. Darker, Nicola O'Connell, Martin Dempster, Christopher D. Graham, Cliodhna O'Connor, Lina Zgaga, Ann Nolan, Katy Tobin, Niamh Brennan, Gail Nicolson, Emma Burke, Luke Mather, Philip Crowley, Gabriel Scally, Joseph Barry

**Affiliations:** 1 Public Health and Primary Care, Institute of Population Health, Trinity College Dublin, Ireland, D24 DH74, Ireland; 2 School of Psychology, Queen's University Belfast, 18-30 Malone Road, Belfast, BT9 5BN, UK; 3 School of Psychology, University College Dublin, Newman Building, Belfield, D04 V1W8, Ireland; 4 Trinity Centre for Global Health, Trinity College Dublin, 7-9 Leinster Street South, Dublin, D02 K104, Ireland; 5 Trinity Institute of Neuroscience, Trinity College Dublin, Lloyd Building, Dublin, D02 PN40, Ireland; 6 Trinity College Library, Trinity College Dublin, Dublin, D02 PN40, Ireland; 7 Quality Improvement, Health Service Executive, Dr Steevens’ Hospital, Dublin, D08 W2A8, Ireland; 8 School of Medicine, University of Bristol, Bristol, Tyndall Venue, BS8 1TH, UK

**Keywords:** Behavioural sciences, psychosocial factors, Public Health, Infectious Disease, Epidemiology, Communications media, public policy, mixed methods

## Abstract

COVID-19 represents a serious challenge to governments and healthcare systems. In addition to testing/contact tracing, behavioural and social responses such as handwashing and social distancing or cocooning are effective tools for mitigating the spread of the disease. Psychological (e.g., risk perceptions, self-efficacy) and contextual factors (government, public health messaging, etc.) are likely to drive these behaviours. Collated real-time information of these indicators strengthens local, national and international public health advice and messaging. Further, understanding how well public health and government messages and measures are understood, communicated via (social) media and adhered to is vital. There are two governments and public health jurisdictions on the island of Ireland, the Republic of Ireland (ROI) and Northern Ireland (NI). This represents an opportunity to explore implications of differing measures and messaging across these two jurisdictions as they relate to COVID-19 on two similar populations. The expert research team are drawn from a range of disciplines in the two countries.

This project has four nested studies:

1. Assessment of key behavioural, social and psychological factors through a large, prospective representative telephone survey of individuals aged over-18 on a weekly basis over eight weeks (n=3072); and conduct qualitative focus groups over the same period.
2. Interrogation of social media messaging and formal media responses in both jurisdictions to investigate the spread of (mis)information.
3. Modelling data from Studies 1 and 2, plotting the psychosocial/behavioural and media messaging information with international, ROI and NI incidence and mortality data. 
4. Conducting an assessment of health policy transfer in an attempt to incorporate the most significant public health and political insights from each jurisdiction.

The CONTAIN project will develop an evidence-based toolbox for targeting public health messaging and political leadership and will be created for use for the anticipated second wave of COVID-19, and subsequently for future epidemics/pandemics.

## Introduction

COVID-19 is a novel, highly contagious coronavirus ^1^, not previously seen in humans, which causes respiratory problems that are potentially fatal ^2^. It is primarily thought to be spread through person-to-person contact via droplets spread by an infected person coughing or sneezing, and through touching of contaminated surfaces. COVID-19 represents a threat to public health of both national and global concern. The first case was reported in December 2019 in Wuhan, China ^3^ with the first case confirmed in Ireland in February 2020 ^4^. At present, there are no specific antiviral drugs against COVID-19 infection, and vaccine roll out has commence in some countries in a staggered way ^5^. Therefore, extensive and intensive measures to reduce person-to-person transmission of COVID-19 are required to effectively control the current pandemic.

Alongside testing and contact tracing, behavioural responses (e.g., handwashing and practicing of safe cough and sneeze etiquette) and social responses (e.g., self-isolation, social distancing and cocooning) are the most effective public health responses to slow the spread of the virus. Psychosocial factors affect the spread of infectious viral diseases, affecting adherence to desired health behaviours and public health measures ^6^.

For example, parallels have been drawn in the social and behavioural health responses to COVID-19 from four decades of the HIV pandemic and Ebola ^7^; these include interpersonal factors such that sustained individual-level behavioural change is challenging to achieve; people can misattribute the physical effects of anxiety as evidence of infection ^8^; community-level factors play a role with regards to the ability for community mobilisation for disease prevention; and there may be cyclical patterns of fear which lead to loss of trust in health services, government and medical public health advice which may lead to disruptions in community cohesion ^9^.

There is a further wealth of research from previous viral outbreaks and also emerging COVID-19 research to inform our understanding of increased risk awareness. Self-reports of protective behaviours predict perceived likelihood of personal infection, rather than transmission ^10^. Likewise in a recent study in South Korea, in the early stages of COVID-19 outbreak, practicing precautionary behaviours were associated strongly with perceived risk and response efficacy behaviours ^11^.

The psychological impact of a new pandemic virus can be considerable. Anxious individuals report lower hygiene but greater social distancing during H1N1 in Hong Kong ^12^. With regards to COVID-19, the World Health Organization (WHO) has warned about the considerable fear, worry and concern in populations at large and among certain groups in particular, such as older adults, care providers and people with underlying health conditions ^13^. Emerging data from Italy during COVID-19 quarantine has demonstrated that female gender, negative emotional affect and detachment were associated with higher levels of depression, anxiety and stress; alongside this, having a family member infected was associated with higher levels of anxiety and stress ^14^.

News coverage in the form of both formal media narratives and social media consumption has itself spread very quickly during the pandemic. Novel epidemics can lead to false beliefs and a lack of knowledge increases fear ^15^. This in turn can influence behaviours. Despite the development of antimicrobial drugs, infectious diseases continue to generate fear, as recently demonstrated by the worldwide epidemics of influenza A (H1N1) in 2009, avian influenza A (H5N1) in 2005–2006, and severe acute respiratory syndrome (SARS) in 2003 ^16^.

The media may be a useful resource in controlling epidemic fear, enabling a bridge between government, science and public opinion. Media coverage can directly affect public risk perceptions, and recent studies have shown that media-triggered public concern may influence health-related personal measures taken during pandemics ^17, 18^. International scientific literature has shown that, in more recent epidemics, media coverage may have had a positive influence on disease perception ^19, 20^. Trust in media organisations and the strength and clarity of public health advice guides behaviours and beliefs ^21^.

The science of public health is rarely incontrovertible and while population health identifies potential solutions to public health threats like COVID-19, politics is ultimately the realm in which public policy decisions are made. Across the world, different responses to the pandemic have demonstrated the complex relationship between public health, science and politics with the potential of the pandemic to both make and destroy political reputations ^22^. When politics rather than careful scientific analysis makes policy for public health, there is generally a cost to citizens, as the international literature in other disease outbreaks has illustrated ^23– 25^. Low compliance with public health messaging has been associated with mistrust of governmental institutions ^26^ pointing to the importance of political leadership in containing the spread of COVID-19.

On the island of Ireland there are two public health and governmental jurisdictions, the Republic of Ireland (ROI) and Northern Ireland (NI). The ROI has a population size of approximately 4.9 million people ^27^ and is governed by the Oireachtas. The Health Service Executive (HSE) is responsible for the provision of health and personal social services including a remit for public health and health protection. It is funded by public money. Northern Ireland (NI) has a population size of 1.8 million people ^28^ and has a devolved government from Westminster in the United Kingdom called the ‘Northern Ireland Assembly’. The Public Health Agency is the regional organisation for health protection and for provision of health and social wellbeing services in NI. The ROI and NI occupy the island of Ireland and share a land border. The context within Ireland therefore represents a unique opportunity to explore the implications of COVID-19 on two very similar populations. The WHO has called for a united international response to the pandemic. Ireland is following European Centre for Disease Control advice; while *“Britain* [including NI] *is taking a different response. It is no longer a member of the EU*” ^29^.

In the current proposed study, the COvid-19 Toolbox for All IslaNd (CONTAIN) project, we will identify relevant, feasible and effective approaches to high acceptance and adherence to public health messages and measures, alongside common psychological drivers of messaging adherence in both individuals’ behaviour and societal discourse to improve understanding and trust during COVID-19 response. The study will further map the policy transfer pathway from multilateral actors to policy communities responding to COVID-19 in ROI and NI in order to gain insight into the role of political leadership in determining the shape of the response to the pandemic in different jurisdictions. This will be mapped alongside epidemiological data of incidence and mortality to develop a toolbox for national and global public health leadership in the immediate term and the predicted second COVID-19 wave. This research aligns with two thematic research priorities in the WHO’s Coordinated Global Research COVID-19 Roadmap 2020: ‘public health’ and ‘media communications’ ^30^.

Further to this, we propose to address four research questions within the CONTAIN study. Firstly, what are the relevant, feasible and helpful behavioural and psychosocial approaches to improving adherence to public health recommendations to COVID-19 within Ireland? Secondly, do differences in measures and messaging affect incidence and mortality? Thirdly, what is the role of the wider media and social media landscape in improving public health understanding and trust? And fourthly, what is the role of political leadership in enabling adherence to public health messaging?

### Protocols

**Figure 1.**
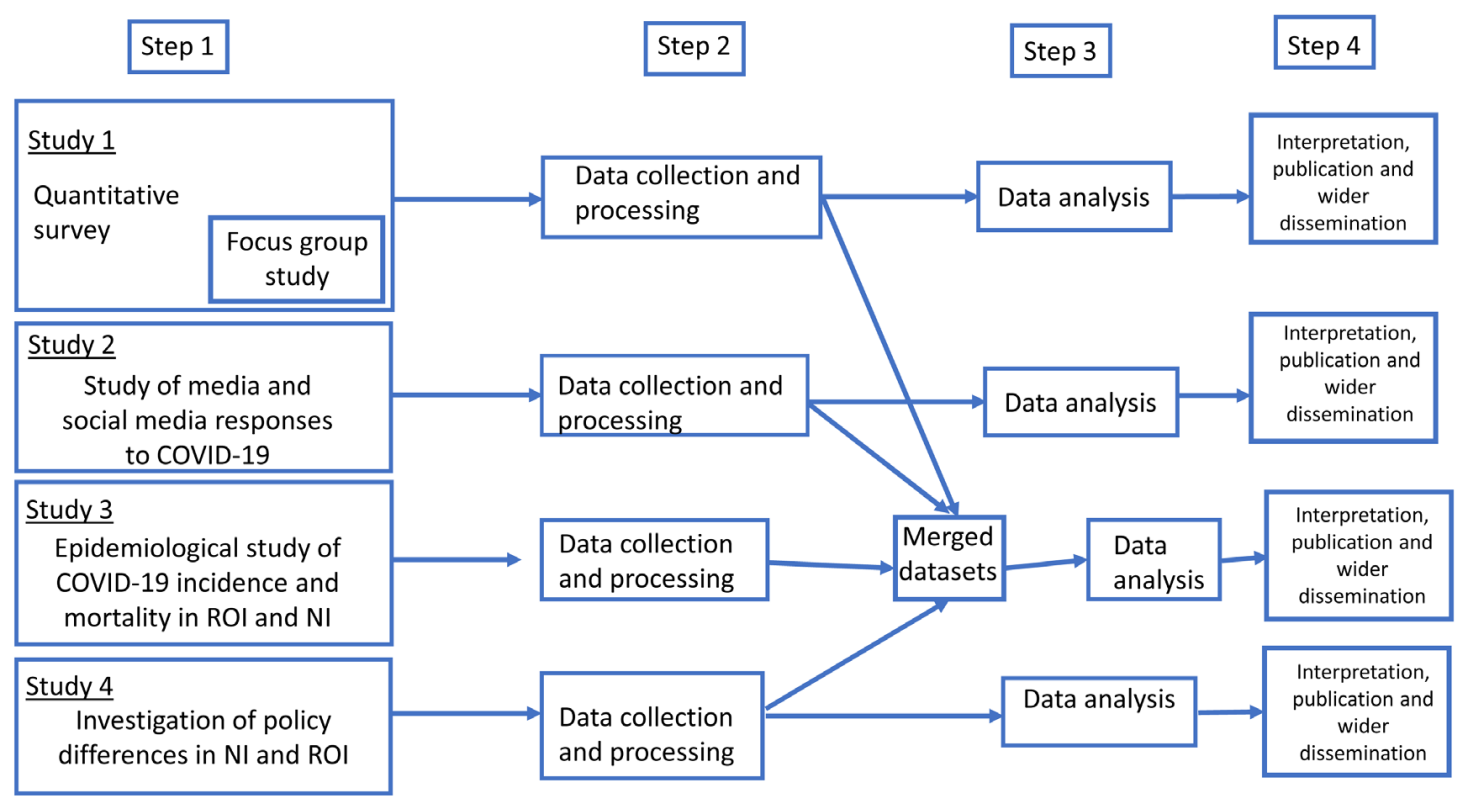
Graph showing study’s mixed method design.

We propose four nested studies using mixed methodologies using a convergent parallel design ^31^ ( Figure 1). Study 1, which entails a quantitative survey and a focus group methodology, has an embedded design ^32^ whereby the weekly high-level results of the survey will inform the questions of the focus group. A convergent parallel design will be used to guide the overall data collection, analyses and interpretation of quantitative and qualitative data arising from this research ^31^. The quantitative and qualitative methods from Studies 1–4 will be considered in the same phase of the research process, each method will carry equal weight, allowing components to be analysed independently initially, with the interpretation of the results together to allow for a comprehensive understanding of the findings as they relate to COVID-19. This will allow for a holistic and multidimensional understanding of the interaction of the factors under observation within this research.

The calendar date of an event, measure, message and incidence/mortality rate will be the unifying unit of analyses across the study as a whole. A timeline matrix will be developed with findings from each component of the study, and updated regularly. This will be assessed for convergence, complementarity or apparent dissonance between factors under observation to relationships and patterns within the data. This design was considered superior to three alternative mixed method approaches: an embedded design whereby one dataset provides a supportive secondary role; an explanatory design whereby qualitative data helps to explain or build upon quantitative results; or an exploratory design where measures or instruments are not available and no guiding theory or framework exists.

The data capture method and analyses process within each individual study is described below.

### Study 1: Psychosocial and behavioural responses to COVID-19

**Figure 2.**
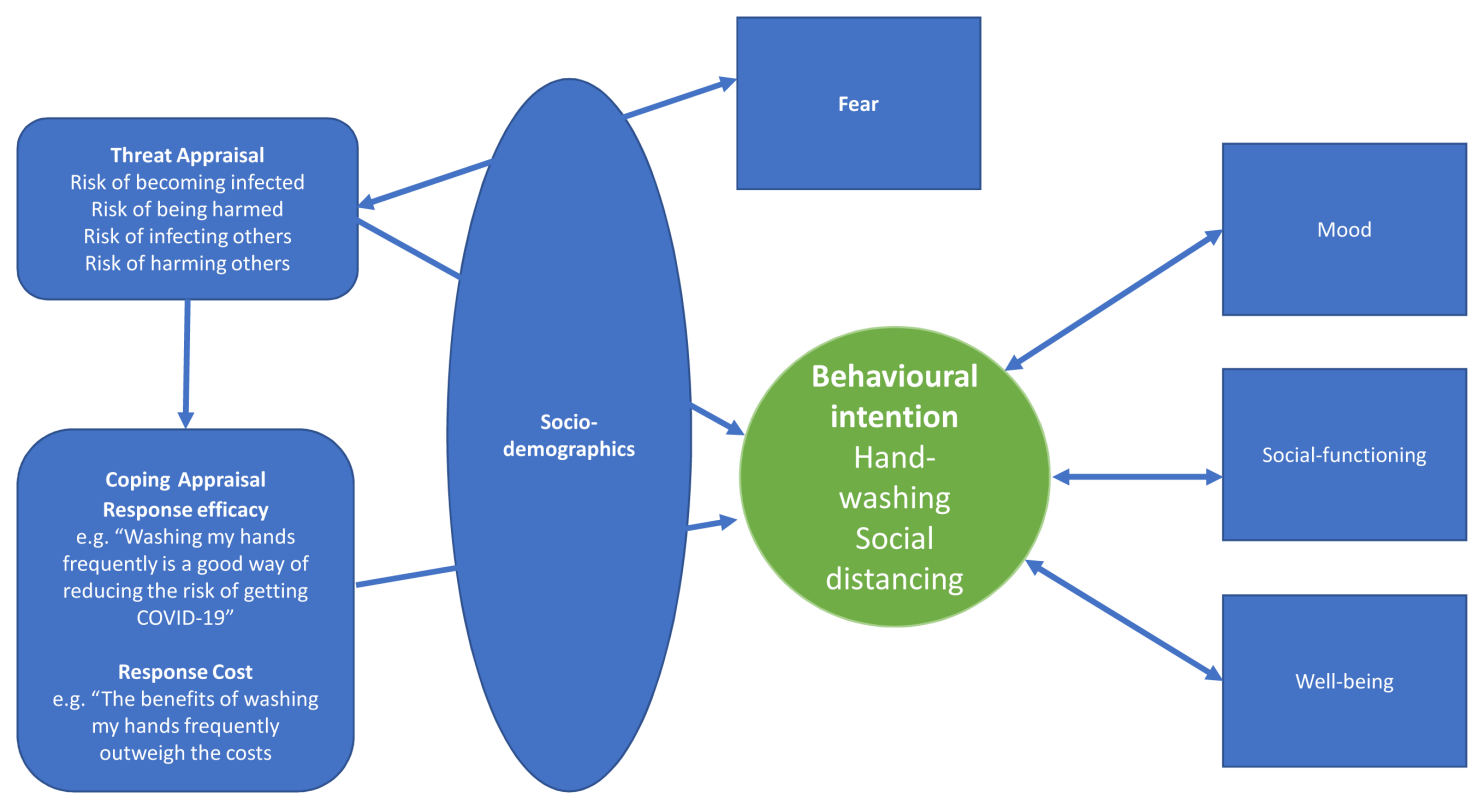
Graph showing application of Protection Motivation Theory to this study.

A large-scale telephone survey will be used to a) delineate the common drivers of adherence to COVID-19 containment behaviours (social distancing, hand-washing), and b) estimate the subsequent impact on well-being of adherence to containment behaviours. Regarding objective a), Protection Motivation Theory (PMT) ^8^ informs the variables we will test as drivers of containment behaviours ( Figure 2). This posits that estimations of threat (e.g., perceived severity/personal vulnerability to threat), and protective responses moderate containment behaviours. Regarding objective b), while containment behaviours should reduce the spread of the virus, it is unknown whether adherence to these behaviours will have a negative effect on well-being (e.g., mood, loneliness), so this will also be explored in Study 1.

Ipsos-MRBI will conduct an all-Ireland telephone survey (see *Extended data*, OSF file for the survey questionnaire ^33^), using a random digit dialling sampling strategy (80% mobile phone; 20% landline) on a weekly basis over eight weeks to assess changes.

The eligibility criteria for survey participation are:

- i) aged 18 years and older;
- ii) able to communicate in English;
- ii) ownership of a landline or mobile phone; and
- iii) residency in ROI or NI

Over the eight-week period, a sample size of n=3072 is required for the survey (approx. 75/25% spilt Republic of Ireland: Northern Ireland, reflective of population distribution). The sample will be weighted to be representative of the population by age, gender and socio-economic class. Survey participants will not receive payment for participation. Survey participants will be different every week; however, they will be sampled in the same way. The primary survey analysis examining the relationship between COVID-19 behaviours and study covariates will be carried out on the full dataset, consisting of n=3072. Longitudinal analysis will be limited to bivariate relationships.

Specific variables to be collected include: threat perceptions, response efficacy, self-efficacy, fear, response cost, social norms, COVID-19 containment behaviours (adapted from Protection Motivation Theory ^34^, depression (PHQ-2 ^35^), anxiety (GAD-2 ^36^), and loneliness ^37^) and demographics (age, gender, SES, location). Based on a significance of 0.05, acceptable error of 5%, and an estimated standard deviation (SD) of 0.5 (categorical comparisons), a sample size of 384 is required for generalisable findings ^9^. The survey will be carried out for descriptive purposes, therefore the sample size is based on the required level of precision, set at 5%.

A weekly focus group will coincide with the survey (see *Extended data* OSF file for an example focus group interview schedule ^33^) and will include 6–8 participants identified and recruited by Ipsos-MRBI. Eligibility criteria are as follows: residency on the island of Ireland; aged 18 years of age and above; ability to communicate in English; and access to conference call software. Ipsos-MRBI staff will phone focus group participants and explain the rationale for the study. If participants express interest, they will be emailed an information leaflet with more information on the study and will receive contact details for the TCD research team. If they decide to participate, they will be required to email a member of the TCD research team or the Ipsos team to give their formal consent to participate. Participants who attend the focus group will receive a €40/£36 gift voucher as payment for their time.

Certain groups will not be sampled, for example people resident in prisons or care homes. In Ireland, those in prisons are not permitted to utilise a mobile phone and do not have routine access to a landline. While sampling institutions would be useful to avoid sampling bias, the study aims to sample the general population rapidly. The study procedure’s emphasis on fast sampling will allow sampling of care homes, where the consent procedure would be more onerous. 

To remain agile to changes in governmental/public health measures/messaging, some items will change weekly to explore issues pertinent that week (e.g., change in restrictions, face masks) and/or to qualitatively explore a specific aspect of the survey (e.g., impact on mental health). A further rationale for the focus groups was for them to run parallel with the weekly telephone surveys to inform any changes required to the interview schedule related to the focus groups. Eligibility may therefore change for specific focus groups. For instance, to explore the effects of COVID-19 on younger people, we may run a group where we restrict participation to those aged 18–24, or amongst those aged over 70 years of age.

Analyses will be conducted using content thematic analyses ^38^. Both the survey instrument and focus group processes will be piloted. The focus group interviews will be conducted via remote video conferencing and will be recorded and transcribed verbatim. Participants will receive a gift card for their participation which will be posted. Field notes will be used to supplement audio and transcriptions. The focus group interview schedule will be revised prior to the next interview and interim survey study results will be assessed to help shape the upcoming week’s interview.

## Data analysis 

### Survey study

The primary analysis will be an examination of the relationship between covariates of demographic variables and PMT variables (gender, age, SES), location, threat perceptions, response efficacy, self-efficacy, fear, response cost, social norms) and the outcome variables relating to COVID-19 behaviours. This will be conducted using regression analysis.

We will conduct longitudinal comparisons, comparing responses each week using independent samples *t* tests, one-way analysis of variance (ANOVA), or chi-square tests where appropriate. In addition, we will compare outcomes in respondents living in NI and ROI, and those living in border regions to people living in non-border regions. Weekly analysis of the surveys will be used to offer exploratory themes, questions and question-related probes for the weekly focus group interviews. Data analyses will be conducted with SPSS or R, and statistical significance will be set at *p* < 0.05 (two-sided).

### Focus group study

Data from focus group interviews will be collected, transcribed and analysed promptly to allow emerging themes to potentially be explored in following interviews ^29^. Thematic analysis will be used ^39^. Data will be coded and analysed independently by two study investigators using NVivo software (QSR International). A codebook will be developed to assist with the coding scheme and data characterisation. The codebook will contain code definitions and will be hierarchical in structure. To improve reliability, a third investigator will resolve any disagreements in coding through discussion. Coding categories will be represented in diagrammatic form.

This study seeks to avoid the weakness inherent in single-method, single-observer studies by adopting a mixed method approach. It will involve rigorous qualitative research including systematic data coding, detailed documentation of analytic decisions and direct quotations from participants to offer readers perspective on the evidence from study findings and conclusions drawn. This will allow for triangulation of findings by the research team ^40^.

### Study 2: Media/social media analysis

The key objective in Study 2 of the CONTAIN project is the examination of news and social media messaging and its societal influence. Inclusion criteria is news and social media content produced in the ROI and NI within a set time frame, which will end in parallel with the conclusion of WP1 telephone survey data collection.

Quantitative modelling will explore the relationships between social/formal media patterns and the epidemiological outcomes described in Study 3. Qualitative analyses will explore the themes present within the media dataset. While such themes may have implications for behaviour, there will not be any attempt to directly correlate these qualitative findings with quantitative indices of behaviour. 

### Data collection

1. A dataset of news media will be constructed via keyword searches of the databases Nexis Advance, Factiva and Bloomberg Business. The databases will be searched using a purposely designed keyword query (coronavirus OR covid* OR SARS-CoV-2), that aims to provide a comprehensive and unbiased sample of media coverage of the pandemic. The search will be restricted to material published in Irish and Northern Irish publications between 1 December 2019 and the end of data collection for Study 1 (estimated 31 July 2020; end-date may be extended if resources permit). The extracted data will represent a master dataset of print media coverage of the pandemic.
2. Social media analysis will be facilitated through purchase of a dataset of Twitter content from a media analytics service (Vicinitas). The company will provide us with all public tweets published between 1 Dec 2020 and 31 July 2020 (end-date may be extended if resources permit), which contain the words or hashtags [covid OR covid19 OR covid-19 OR covid_19 OR coronavirus OR corona OR covid19ireland OR coronavirusireland OR covid19northernIreland OR covid19NI OR covid19UK] and are geolocated to the island of Ireland.
3. Additional insights into print, broadcast and social media coverage will be provided by the NewsWhip Analytics service. Purposely created dashboards will produce summary data on the online content (e.g. web articles, Facebook pages) that generated most social media engagement (platforms include Facebook). Data will be filtered by keyword, date and location and used in the analysis.

### Data analysis

1. The complete master datasets of print and social media will be analysed as follows:

- Frequency analysis will track the quantity of coverage across the time period. This will be interpreted through cross-reference with Study 1 longitudinal survey data and Study 3 epidemiological data.
- Automated analysis of media content will identify patterns using word frequency, word association and sentiment analysis tools. Analysis will seek to identify meaningful differences in content across time (including time periods differentiated by intensity of incidence/mortality), geographical areas (Republic of Ireland vs Northern Ireland, and more fine-grained regional differences if sufficient geo-identified data is available), and source type (social media vs formal media, broadsheet vs tabloid, national vs regional).

2. More granular analysis of specific aspects of media discourse will be performed on smaller subsets of the master datasets. Based on keyword searches and/or date parameters of interest, relevant media data will be extracted and exported into NVivo for manual analysis. To allow agility to and integration with Studies 1, 3 and 4, the full range of these sub-analyses has not been predefined and will be determined as results emerge. For instance, if the survey reveals low adherence to a specific public health recommendation, or the epidemiological analysis identifies a particularly key date in the trajectory of the pandemic, corresponding media data can be identified as required. Additional overarching concerns will include:

- Representations of science and medical expertise;
- Expression of dissensus from public health advice;
- Emotional tone of discourse; and
- Mental health implications of pandemic.

Content analysis will identify the discursive features that characterise the extracted datasets. Content analysis aims to reduce large quantities of text into their salient categories of meaning using explicit coding procedures ^41^. Study-specific coding schemes will be developed through both inductive and deductive techniques, to allow analysis that responds to both pre-existing research questions and emergent features of the data. Coding will be performed using NVivo and subject to inter-coder reliability checks to ensure credibility and consistency ^42^. 

NewsWhip Analytics will provide data to complement the above two forms of analysis. Dedicated keyword-based dashboards will be built to track the online content relevant to a particular topic that generated most social media engagement. Results will be filtered by date, location and source of material to allow cross-reference with the print media and Twitter datasets.

### Study 3: Merging data against epidemiological data

The primary objective to study three is to map the individual outlook and behaviours data as captured in the survey (Study 1) and key media content (Study 2) to epidemiological data, to examine whether these reflect COVID-19 spread, or *vice versa*, whether disease incidence and mortality impact on the media and sentiment. Official sources will be searched to gather data on incidence and mortality in ROI (e.g., Health Protection Surveillance Centre) and NI (e.g., Northern Ireland Statistics and Research Agency) and also from the relevant Departments of Health in the two jurisdictions. Study 3 involves the mapping of publicly available numbers of new cases (and incidence) and mortality rates in both ROI and NI. We will also seek data on age, gender and other demographic information of cases and deceased, and include in analysis if possible. More granular data on a county level will also be sought and similar analysis will be undertaken, covering smaller geographical regions to see if we can identify different patterns within ROI and NI – for example, in border counties.

To achieve this objective on weekly basis and in real time, survey data will be summarised and mapped against incidence/mortality in ROI and NI separately. Time series regression will examine whether changes in outlook and behaviours are linked with epidemiological data. The incubation period/time to death or recovery will be taken into account as much as possible. Alongside this key (social) media content and official public health messages (e.g. social distancing) and restrictions (e.g. travel ban) will be mapped onto key epidemiological data, retrospectively from Feb 1 ^st^ 2020) and prospectively during the project life for both regions. We will attempt to identify content, policies and communications that were useful in slowing the disease spread. Differences ** between ROI and NI will be analysed. Finally, key policies identified in Study 4 will be coded by date, and mapped onto these data, and where possible the uptake of key policies.

The intention of the analysis is not to determine whether behaviours are statistically significantly associated with the epidemiological data, but rather to describe trends in both and see how they relate. In addition, Study 3 will attempt to use national epidemiological data during the pandemic, together with national media and social media data on the island of Ireland at the same time, amounting to hundreds of thousands of observations each day and will allow an examination of how they varied in time. 

### Study 4: Political leadership for COVID-19

The primary objective of Study 4 is to chart policy-transfer from multilateral actors to policy communities responding to COVID-19 in the ROI and NI in order to understand the role of political leadership in determining the response in each jurisdiction. An assessment of the extent to which political leaders in the ROI, NI and the United Kingdom have been engaging with multilateral “transfer agents” will be examined in order to understand the extent to which global health policy objectives are altered at the point of implementation to take account of local preferences. This study will further consider the role of the Good Friday Agreement, specifically North-South co-operation on the island of Ireland, and Brexit, the withdrawal of the United Kingdom from the European Union, in shaping the response to COVID-19. 

The parameters of the study define multilateral guidance in this instance as the regular COVID-19-specific publications and guidance documents published by the European Centre for Disease Prevention and Control (ECDC) from 17 ^th^ January 2020 to the end of data collection for Study 1 (estimated 31 July 2020; end-date may be extended if study resources permit). Study 4 will adopt the indicators contained in the authoritative codebook for the Oxford COVID-19 Government Response Tracker, which aims to track and compare policy responses to COVID-19 from around the world ^43^. The Tracker dataset contains 17 indicators organised into four policy groups, however due to the short duration of the CONTAIN project three of the four policy groups will be utilised (see Table 1):

- C - containment and closure policies
- H - health system policies
- M - miscellaneous policies

**Table 1.** Oxford COVID-19 Government Response Tracker Codes (43) applied to CONTAIN Study 4 data collection.

Containment and closure policies			
OxTracker. CONTAIN ID	Name	Description	Codes
C1	C1_School closing	Record closings of schools and universities	0 = aligned (with multilateral guidance) 1 = not aligned (with multilateral guidance)
C2	C2_Workplace closing	Record closings of workplaces	0 = aligned 1 = not aligned
C3	C3_Cancel public events	Record cancelling public events	0 = aligned 1 = not aligned
C4	C4_Restrictions on gatherings	Record limits on private gatherings	0 = aligned 1 = not aligned
C5	C5_Close public transport	Record closing of public transport	0 = aligned 1 = not aligned
C6	C6_Stay at home requirements	Record orders to "shelter-in-place" and otherwise confine to the home	0 = aligned 1 = not aligned
C7	C7_Restrictions on internal movement	Record restrictions on internal movement between cities/regions	0 = aligned 1 = not aligned
C8	C8_International travel controls	Record restrictions on international travel **Note: this records policy for foreign travellers, not citizens**	0 = aligned 1 = not aligned
**Health system policies**			
OxTracker CONTAIN ID	Name	Description	Codes
H1	H1_Public information campaigns	Record presence of public info campaigns	0 = aligned 1 = not aligned
H2	H2_Testing policy	Record government policy on who has access to testing Note: this records policies about testing for current infection (PCR tests) not testing for immunity (antibody test)	0 = aligned 1 = not aligned
H3	H3_Contact tracing	Record government policy on contact tracing after a positive diagnosis Note: we are looking for policies that would identify all people potentially exposed to Covid-19; voluntary bluetooth apps are unlikely to achieve this	0 = aligned 1 = not aligned
**Miscellaneous policies**			
OxTracker CONTAIN ID	Name	Description	
M1	M1_Wildcard	Record policy announcements that do not fit anywhere else (Study 4 will apply cross-border collaboration to the wildcard policy group)	

The Oxford COVID-19 Government Response Tracker captures the most stringent policy in a limited geographic area or sector and a binary flag variable is used to denote this limited scope. Consequently, the devolved legislature of Northern Ireland is not differentiated from the United Kingdom in the dataset. CONTAIN’s Study 4 will consequently add value to the Covid-19 Government Response Tracker by using qualitative research methods to mine policy response data from the World Health Organisation/European Commission and the European Observatory on Health Systems and Policies (EOHS&P) COVID-19 ‘Health System Response Monitor for the United Kingdom and Ireland’ ^44^ other key policy sources in each jurisdiction using 12 indicators from three of the Tracker’s policy groups.

### Data collection

1. Twelve indicators from the Oxford COVID-19 Government Response Tracker codebook in three policy groups (C – containment and closure policies; H- health system policies; M – miscellaneous policies recoded to reflect NI/ROI cross-border collaboration) will direct qualitative data collection from:

1.1 ECDC COVID-19 related publications and guidance documents from 17 ^th^ January 2020;
1.2 World Health Organisation/European Commission and the European Observatory on Health Systems and Policies (EOHS&P) COVID-19 ‘Health System Response Monitor for the United Kingdom and Ireland’
1.3 Gaps in the data will be filled by interrogation of minutes and policy statements made available by the Irish Government and the National Public Health Emergency Team ^45^; the Northern Ireland Assembly ^46^ and the UK Government Scientific Advisory Group for Emergencies (SAGE) ^47^ and slides, datasets and transcripts to accompany coronavirus press conferences published by the UK Government.

This data will represent a master data set from which cross-case and within-case analysis will identify points of alignment and divergence between ECDC COVID-19 guidance and the policy responses in NI and the ROI across 12 indices.

### Data analysis

1. The policy response data for each jurisdiction and policy response area will be mapped in Excel using a conceptually clustered matrix. Study 4’s thematic codes will reflect those of the Oxford COVID-19 Government Response Tracker in three policy groups (see Table 1).
2. Schemes will be developed through primarily deductive techniques with some inductive processes to enable research questions to be answered with emergent and outlier themes. Cross-case analyses will highlight points of policy alignment and divergence with multilateral actors and similarity and difference in policy responses for COVID-19 on the island of Ireland. Cross-case analysis is a method that involves the in-depth and systematic exploration of similarities and differences across cases in order to facilitate empirical generalisability and theoretical predictions.
3. The World Bank’s ‘Problem-Driven Governance and Political Economy Analysis : Good Practice Framework’ ^48^ will provide a second layer of data analysis in order to examine the socio-political and structural context, the agents, incentives and power-relationships between stakeholders that impact on the policy response for COVID-19 in each jurisdiction.
4. Study 4 data will be mapped with the findings of studies 1-3 and the Political Economy Analyses ^49^ will reflect findings from the CONTAIN study.

### Reporting frameworks

We will use the Strengthening the Reporting of Observational Studies in Epidemiology (STROBE) Statement ^50^ and also the Consolidated Criteria for Reporting Qualitative Research (COREQ) ^51^, where appropriate.

### Ethical approval

This research has been approved by the National Research Ethics Committee for COVID-19 research. Reference number: 20-NREC-COV-037.

### Plans for dissemination

We will integrate findings from each study within a study website dedicated to the CONTAIN project, hosted on the lead academic institutions website. This website will be a tool for academics, public health officials and public health policy makers. This website will be updated regularly as the study develops and will host links to each of the open access articles we publish.

We will seek to maximise the availability of research data produced over the course of this project in recognition that sharing data helps public health researchers and policymakers build on existing knowledge and make discoveries that can improve our response to this pandemic. We commit to the ‘Statement on data sharing in public health emergencies’ principles (Wellcome Trust, 2016). All relevant research findings from the CONTAIN project will be shared immediately with the WHO upon submission of papers to journals. We will share data with senior public health officials in the National Health Service (NHS) and the Health Service Executive (HSE) in NI and ROI respectively as well as the ministers for health in both jurisdictions. We will provide policy briefings to senior officials in the HSE and Northern Irish NHS, both Departments of Health, and we will adapt these briefings to ensure lay versions are available for public and patient audiences. Press releases will be issued and coordinated via TCD’s Communication Office. We will conduct webinars for relevant stakeholder groups such as healthcare, policymakers and public bodies.

FAIR data management principles will be followed, and any resulting papers will be published as open access, immediately accessible journals (for example, the International Journal of Public Health) and will be deposited in TCD TARA, an open access repository which is OpenAIRE- and FAIR-compliant. Data will be archived in trusted sites including the Irish Social Science Archive, the Irish Qualitative Data Archives, and Zenodo.

Data sharing agreement (project-wide) will be finalized, participant-training in GDPR and national health regulations assured, and a Data Impact Assessment Form completed and assessed by TCD’s Data Protection Officer. An embedded HRB GO-FAIR data steward will ensure HRB DMP maintenance/reporting and FAIR RDM. Data, both qualitative and quantitative (spreadsheets, transcripts/audio, text, databases), will be stored on encrypted computers, with daily back-ups to external hard drives and regular downloads to a secure external location. Data sharing during the project will be via access to a shared institutional drive with secure cross-institutional data-sharing provided via institutional computing services (backed up nightly to strict security protocols).

FAIR data management will be achieved through agreed data description, file naming conventions and metadata standards, DOI assignment and machine-actionable shared data (if possible), anonymised (where necessary) via trusted repositories with Creative Commons licencing.

## Conclusion

The findings of the CONTAIN study will be relevant to policy and practice, in particular to public health planning in anticipation of a COVID-19 ‘second’ wave. This study will help Governments and public health agencies to package guidelines in a way that will be most effective in ensuring recommended behaviours are adopted widely by the general public. The survey results and interviews will serve to contextualise our media, epidemiological and policy findings and may help in the design of subsequent studies and public health interventions to support responses to this and future pandemics.

## Data availability

### Underlying data

No underlying data are associated with this manuscript.

### Extended data

Open Science Framework: CONTAIN. https://doi.org/10.17605/OSF.IO/VD83H ^33^.

This project contains the following extended data:

- Extended_data_Focus Group Interview Schedule (DOCX). (Interview schudeule and questions to be used to focus groups.)
- Extended Data_Ipsos MRBI survey study (DOCX). (Blank survey to be used during telephone interviews.)

Extended data are available under the terms of the Creative Commons Attribution 4.0 International license (CC-BY 4.0).
