# Supplementary material for: Study protocol for the COvid-19 Toolbox for All IslaNd (CONTAIN) project: A cross-border analysis in Ireland to disentangle psychological, behavioural, media and governmental responses to COVID-19
Source: HRB Open Res. 2021 Feb 15;3:48. Originally published 2020 Aug 3. [Version 2] doi: 10.12688/hrbopenres.13105.2 (PMC7898359; doi:10.12688/hrbopenres.13105.2)
Supplement: Peer review history

# Peer review: Reviewer response for version 2

**DOI:** 10.21956/hrbopenres.14393.r28909

**Published:** 2021-02-19

**Authors:** Kim Lavoie

**Affiliations:** 1 Department of Psychology, University of Quebec at Montreal, Montreal, Canada

I am satisfied with the responses of the authors and approve this version of the manuscript.

Is the study design appropriate for the research question?

Partly

Is the rationale for, and objectives of, the study clearly described?

Yes

Are sufficient details of the methods provided to allow replication by others?

Partly

Are the datasets clearly presented in a useable and accessible format?

Not applicable

Reviewer Expertise:

Behavioural epidemiology, psychosocial and behavioural determinants of disease, behavioural medicine, behaviour change, behavioural interventions, behavioural intervention development and testing, disease prevention, adherence, motivation, adherence to COVID-19 prevention measures, policy communication strategies.

I confirm that I have read this submission and believe that I have an appropriate level of expertise to confirm that it is of an acceptable scientific standard.

# Peer review: Reviewer response for version 1

**DOI:** 10.21956/hrbopenres.14208.r28511

**Published:** 2021-01-22

**Authors:** Kim Lavoie

**Affiliations:** 1 Department of Psychology, University of Quebec at Montreal, Montreal, Canada

**Summary**

 This was a manuscript describing a protocol for the CONTAIN Study, which aims to develop a COVID-19 evidence-based toolbox to improve public health messaging among political leadership in Ireland in anticipation of the 2 ^nd^ wave of the pandemic. The protocol describes 4 nested studies:

1. An assessment of behavioural, social and psychological factors associated with behavioural adherence to COVID-19 prevention measures and their impact on well-being, using a large prospective telephone survey with weekly assessments of individuals aged 18 and over, as well as focus groups for qualitative analyses;
2. An interrogation of social media messaging and formal mainstream media responses in ROI and NI to determine its societal influence;
3. A epidemiological modelling study using data from study 1 and 2, plotting the psychosocial and behavioural and media messaging information with international, ROI and NI COVID-19 incidence and mortality rates; and
4. An assessment of the role of political leadership, local political ideology and key events in shaping government responses to the pandemic in ROI and NI, and to determine if global health policy objectives are altered at the point of implementation to take local preferences into account.

**Assessment:**

- Overall, this was a very interesting, innovative and timely manuscript describing the protocol for 4 nested studies, with the aim of improving public health messaging to help improve public policy adherence to COVID-19 prevention measures. Addressing the behavioural aspects of disease transmission has been, and will continue to be, among the THE most important interventions to overcome the pandemic. Even when vaccines are more widely available, the success of vaccination programs will ultimately depend on people’s willingness to get vaccinated – another behavioural issue that will likely be heavily impacted by media messaging. The fact that behavioural research has received so little support worldwide (relative to basic biomedical research) is both short-sighted and counter-productive. The overall project is therefore deemed important and timely, and is expected to generate actionable data for the people of ROI and NI that may also be transferable to other nations.
- The manuscript was very well written and organized. There were 4 studies to describe and the authors provided sufficient detail with which to assess the project and quality of the manuscript.
- The rationales for the 4 studies was well described and well argued. The project was grounded in an appropriate behavioural theory (e.g., Protection Motivation Theory) which is appropriate. Assessing the role of media messaging (social and mainstream) as well as the role of political leadership in enabling adherence to prevention measures is judged to be novel and innovative.
- Overall, both the quantitative and qualitative methods for the 4 studies were clearly described and generally appropriate. There is some concern about the 8 week sampling window, and how data collected will be strongly influenced by the state of the pandemic and policies and messaging during that time period. I have also described some concerns about aspects of the methodologies, and included some questions and suggestions below for the authors to consider as they move forward with this important project, presented by project:
- **Study 1:**
  - Regarding eligibility criteria, have the authors considered potentially excluding those residing in institutions (e.g., senior’s care homes, prisons) – whose determinants of adherence to prevention measures may not be the same as those living in the general population (i.e., they may be more restricted due to institutional regulations)?
  - Though the authors presented a sample size estimate with a power analyses, they did not specify on which outcome(s) they based their estimate on. The high number of predictor variables and outcome variables suggests a sample size of 384 seems low in terms of providing having adequate power and to have adequate representation of individuals from ROI and NI. Even though they will have over 3000 surveys by the end of the 8 weeks, they will have less than 100 participants (25%) from NI, which may have limited generalizability. In the analyses section, they say they will compare outcomes in those from ROI and NI, and those living in border regions to those not – what is the sample representation in these regions? With less than 400 participants, and no data on how many of the 384 are expected to live in border regions, it is unclear if this analysis will be feasible. In short, the power analysis and sample size estimate was not compelling and may sell the study short.
  - Weekly focus groups are proposed to run in parallel to the survey study, though the rationale for this was not clear in terms of what additional data or information they expected to collect using qualitative FG. Is this intention just to collect the same information in 2 ways? This could be clarified.
- **Study 2: **
  - The aim of this study seemed to be to examine the impact of social media messaging and formal mainstream media responses in ROI and NI to determine their societal influence, yet the outcomes for this study were not clearly defined. Do you intend to link media messaging themes to behaviours? This could be clarified.
- **Study 3:**
  - Similar to above, my main concern with the feasibility of study 3 is related to sample size. The authors propose to examine the association between the behavioural data collected in study 1 and media data collected in study 2 with epidemiological case and mortality data (and how this may be impacted by sociodemographic variables and if individual live in border regions). If this is the case, it is rather ambitious given their sample size of 384, so they need to demonstrate that they have the sample size necessary to do so (there was no sample size estimate or power analysis presented in this study description).
- **Study 4:**
  - One of the aims is to examine the role of local political ideology and key events in shaping government responses to the pandemic. What was not clear is how these two variables (political ideology and key events) were defined and how they will be measured. This could be clarified.
- A strength of the protocol and manuscript is that the authors plan to use appropriate reporting frameworks for their publications, and they have obtained ethical approval for the project.
- Plans for dissemination were also well described, comprehensive and appropriate.
- They will use FAIR data management principles and aim to publish in open access journals to increase knowledge transfer, which is appropriate.

Is the study design appropriate for the research question?

Partly

Is the rationale for, and objectives of, the study clearly described?

Yes

Are sufficient details of the methods provided to allow replication by others?

Partly

Are the datasets clearly presented in a useable and accessible format?

Not applicable

Reviewer Expertise:

Behavioural epidemiology, psychosocial and behavioural determinants of disease, behavioural medicine, behaviour change, behavioural interventions, behavioural intervention development and testing, disease prevention, adherence, motivation, adherence to COVID-19 prevention measures, policy communication strategies.

I confirm that I have read this submission and believe that I have an appropriate level of expertise to confirm that it is of an acceptable scientific standard, however I have significant reservations, as outlined above.

# Response

**Published:** 2021-02-04

**Authors:** Nicola O'Connell

**Affiliations:** Trinity College Dublin, Ireland

Thank you for your comments. Please see each reviewer comment below and our associated response: 

1. Reviewer comment Study 1: Regarding eligibility criteria, have the authors considered potentially excluding those residing in institutions (e.g., senior’s care homes, prisons) – whose determinants of adherence to prevention measures may not be the same as those living in the general population (i.e., they may be more restricted due to institutional regulations)?

1. Author Response (CD/NOC): The reviewer raises an interesting point. Participants in Study One were contacted through random digit dialing on the telephone. In Ireland those in prisons are not permitted to utilise a mobile phone and do not have access to a landline in the usual sense of the term.

 While we agree that sampling those in institutions would be useful, this study aimed to sample the general population rapidly. The study procedure, which involved quick and rapid weekly sampling, would not have allowed us to sample care homes which would have involved a more onerous consent process and would have delayed the role out of the survey. We did however conduct a qualitative focus group with the over-70s to assess the experience of older members of the community. We hope this has given some sense of the lived experience of those who are more vulnerable. 

 See update in Line 167, p. 11

2. Reviewer Comment Study One: Though the authors presented a sample size estimate with a power analyses, they did not specify on which outcome(s) they based their estimate on. The high number of predictor variables and outcome variables suggests a sample size of 384 seems low in terms of providing having adequate power and to have adequate representation of individuals from ROI and NI. Even though they will have over 3000 surveys by the end of the 8 weeks, they will have less than 100 participants (25%) from NI, which may have limited generalizability. In the analyses section, they say they will compare outcomes in those from ROI and NI, and those living in border regions to those not – what is the sample representation in these regions? With less than 400 participants, and no data on how many of the 384 are expected to live in border regions, it is unclear if this analysis will be feasible. In short, the power analysis and sample size estimate was not compelling and may sell the study short.

2. Author Response (KT): The primary survey analysis examining the relationship between COVID-19 behaviours and study covariates will be carried out on the full dataset, consisting of n=3072. Longitudinal analysis will be limited to bivariate relationships.

 Regarding the query on “ *they did not specify which outcome(s) they based their estimate on*”. This survey is carried out for descriptive purposes, therefore the sample size is based on the required level of precision, set at 5%.

 See Line 146 and Line 154 (p.10) for updated response in text.

3. Reviewer Comment Study One: Weekly focus groups are proposed to run in parallel to the survey study, though the rationale for this was not clear in terms of what additional data or information they expected to collect using qualitative FG. Is this intention just to collect the same information in 2 ways? This could be clarified.

3. Author Response (CD): The rationale was for the focus groups and the telephone survey to run parallel so as to take the interim analyses of the telephone surveys to inform any changes required to the interview schedule relating to the focus groups.

 See Line 176, p. 11

4. Reviewer Comment Study Two: The aim of this study seemed to be to examine the impact of social media messaging and formal mainstream media responses in ROI and NI to determine their societal influence, yet the outcomes for this study were not clearly defined. Do you intend to link media messaging themes to behaviours? This could be clarified.

4. Author Response (COC): Quantitative modelling will explore the relationships between social/formal media patterns and epidemiological outcomes. Qualitative analyses will explore the themes present within the media datasets. While such themes may have implications for behaviour, there will not be any attempt to directly correlate these qualitative findings with quantitative indices of behaviour. 

 See Line 220, p. 12 for updated text

5. Reviewer Comment Study Three: Similar to above, my main concern with the feasibility of study 3 is related to sample size. The authors propose to examine the association between the behavioural data collected in study 1 and media data collected in study 2 with epidemiological case and mortality data (and how this may be impacted by sociodemographic variables and if individual live in border regions). If this is the case, it is rather ambitious given their sample size of 384, so they need to demonstrate that they have the sample size necessary to do so (there was no sample size estimate or power analysis presented in this study description).

5. Author Response (LZ): The intention of the analysis is not to determine whether behaviours are statistically significantly associated with epi data, but rather to describe trends in both and see how they relate. In addition, Study 3 will attempt to use national epi data during the pandemic together with national media and social media data on the island at the same time, amounting to hundreds of thousands of observations each day and examine how they varied in time.

 Updated text on Line 307, p. 14

6. Reviewer Comment Study 4: One of the aims is to examine the role of local political ideology and key events in shaping government responses to the pandemic. What was not clear is how these two variables (political ideology and key events) were defined and how they will be measured. This could be clarified.

 6. Author Response (AN): The following sentence has been inserted to improve clarity: “ *This study will further consider the role of the Good Friday Agreement, specifically North-South co-operation for health on the island of Ireland, and Brexit, the withdrawal of the United Kingdom from the European Union, in shaping the response to COVID-19.”*

 Line removed and replaced with the above. See Line 320, p. 14

# Peer review: Reviewer response for version 1

**DOI:** 10.21956/hrbopenres.14208.r28095

**Published:** 2020-11-26

**Authors:** Gerry Molloy

**Affiliations:** 1 School of Psychology, National University of Ireland Galway, Galway, Ireland

This is an ambitious programme of work for a 6 month study across the two jurisdictions in Ireland that aims gather a comprehensive range of quantitative and qualitative data form a variety of sources. The four nested studies using a convergent parallel mixed methods design have the potential to provide useful insights from the early period of the pandemic that can guide to inform future public health responses to COVID-19 in both jurisdictions. The all-Ireland data collection, the wide-range of leading experts on the study team and the diversity and integration of the data sources are particular strengths of this programme of work. Some minor suggestions are outline below for any revision of this protocol paper.

1. It would be good to specify that 8 weeks of n=384 will produce the n=3072 sample and to state whether each week will be a 75/25% spilt Republic of Ireland: Northern Ireland i.e. Does this mean that approximately 96 people from Northern Ireland and 288 from RoI were recruited in each wave?
2. It would be good to add some detail on what the power analysis is based on e.g. what is the categorical comparison-is it jurisdiction? Is this calculation based on 80% statistical power?
3. For study 2 it would be good to summarise the form of the output of these analyses at the end of this section. For example, does this produce a set of themes as in a thematic analysis or is there another way to describe what this analysis produces.

Is the study design appropriate for the research question?

Yes

Is the rationale for, and objectives of, the study clearly described?

Yes

Are sufficient details of the methods provided to allow replication by others?

Yes

Are the datasets clearly presented in a useable and accessible format?

Not applicable

Reviewer Expertise:

Health Psychology and behavioural medicine.

I confirm that I have read this submission and believe that I have an appropriate level of expertise to confirm that it is of an acceptable scientific standard.

# Response

**Published:** 2020-11-30

**Authors:** Nicola O'Connell

**Affiliations:** Trinity College Dublin, Ireland

Thank you for your comments and suggestions for improvement of the study. Below we outline our responses to the three points raised:

 1. It would be good to specify that 8 weeks of n=384 will produce the n=3072 sample and to state whether each week will be a 75/25% spilt Republic of Ireland: Northern Ireland i.e. Does this mean that approximately 96 people from Northern Ireland and 288 from RoI were recruited in each wave?

**Response**: Yes, we confirm that over the course of the eight weeks of the survey, a sample of approximately 384 people will be sampled each week in order to produce the overall sample size of 3072. Each week there will be a ratio split of 75:25 split between the Republic of Ireland and Northern Ireland. This means that while we will aim for a sampling targeting of 96 people in NI and 288 in ROI, if participants from one jurisdiction are over- or under-sampled one week, this will be corrected the following week so that by the end of eight weeks, the 75:25 split will be achieved.

 2. It would be good to add some detail on what the power analysis is based on e.g. what is the categorical comparison-is it jurisdiction? Is this calculation based on 80% statistical power?

**Response: **This survey is carried out for descriptive purposes, therefore the sample size is based on the required level of precision, set at 5%. It is anticipated that a comparison of ROI/NI outcomes will play a key role in the analysis, thus the sample size was estimated to allow for this comparison.

 3. For study 2 it would be good to summarise the form of the output of these analyses at the end of this section. For example, does this produce a set of themes as in a thematic analysis or is there another way to describe what this analysis produces.

**Response: **Coding will be performed using NVivo and subject to inter-coder reliability checks to ensure credibility and consistency (42). Outputs of these analyses will comprise narrative descriptions (with supporting quotations) of the key thematic categories identified by the analysis.****
